# The intestinal and biliary microbiome in autoimmune liver disease—current evidence and concepts

**DOI:** 10.1007/s00281-022-00936-6

**Published:** 2022-05-10

**Authors:** Timur Liwinski, Melina Heinemann, Christoph Schramm

**Affiliations:** 1 Department of Medicine, University Medical Center Hamburg-Eppendorf, Hamburg, 20246 Germany; 2 Martin Zeitz Center for Rare Diseases, University Medical Center Hamburg-Eppendorf, Hamburg, 20246 Germany; 3 Hamburg Center for Translational Immunology, University Medical Center Hamburg-Eppendorf, Hamburg, 20246 Germany

**Keywords:** Intestinal barrier, Liver disease, Microbiome, Microbiota, Autoimmunity, Autoimmune hepatitis, Primary biliary cholangitis, Primary sclerosing cholangitis

## Abstract

Autoimmune liver diseases are a group of immune-mediated liver diseases with three distinct entities, including autoimmune hepatitis, primary biliary cholangitis, and primary sclerosing cholangitis. The interplay of genetic and environmental factors leads to the breakdown of self-tolerance, resulting in hyper-responsiveness, and auto-aggressive immune activation. Emerging evidence links autoimmune liver diseases with alterations of the commensal microbiome configuration and aberrant immune system activation by microbial signals, mainly via the gut-liver axis. Thus, the microbiome is a new frontier to deepen the pathogenetic understanding, uncover biomarkers, and inspire innovative treatments. Herein, we review the current evidence on the role of the microbiome in autoimmune liver diseases from both clinical and basic research. We highlight recent achievements and also bottlenecks and limitations. Moreover, we give an outlook on future developments and potential for clinical applications.

## Introduction

The term autoimmune liver disease (AILD) comprises three different idiopathic progressive disorders: primary biliary cholangitis (PBC), primary sclerosing cholangitis (PSC), and autoimmune hepatitis (AIH). The etiologies are unexplained, and the pathogenesis is poorly known [1].

AIH features inflammation with persistent chronic activity and/or flares; hypergammaglobulinemia and a variety of circulating autoantibodies are frequently observed [2]. PBC is characterized by inflammation of the small intrahepatic bile ducts, resulting in bile duct destruction and liver fibrosis; the vast majority of patients display circulating antimitochondrial antibodies (AMAs). PBC shows a strong female preponderance with approximately a 9:1 female-to-male ratio [3]. PSC is a cholestatic liver pathology marked by continuous inflammation, fibrosis, and destruction of intrahepatic and/or extrahepatic bile ducts [4].

Evidence strongly suggests that AIH, PBC, and PSC are heterogeneous and complex disorders with underlying genetic and environmental risk factors [5–8]. However, the etiology of these illnesses remains unknown, and thus, effective therapies are lacking [9]. Patients suffering from end-stage liver disease are treated with liver transplantation [10].

The liver receives a wealth of signals from the gut in the form of nutrients, antigens, hormones, and other molecules. The portal circulation facilitates this link, for which the term “gut-liver axis” has been coined [11]. Akin to all mammals, the human gut harbors a complex community of predominantly bacterial microbes and collective genomes (termed the microbiome) [12]. Understanding the interchange between the human microbiome and disease, including obesity, cancer, inflammatory bowel disease, autoimmune diseases, and neuropsychiatric diseases, is growing rapidly [12]. Advances in the throughput and efficiency of DNA sequencing of microbial genomes, complemented by analysis of transcriptomes, proteomes, metabolomes, and mechanistic experiments in animal models, have tremendously improved our ability to understand the composition and function of the microbiome in health and disease [13]. Liver products fundamentally influence the gut microbiota composition and gut barrier integrity, whereas intestinal factors govern hepatic bile acid synthesis, glucose, and lipid metabolism [14]. The link between the microbiome, gut, and liver is widely implicated in the pathogenesis of liver diseases and is increasingly the focus of medical research [15]. Recent studies have established a link between altered intestinal and biliary microbiota and autoimmune liver diseases, which is the subject of the present review. However, the research community has just begun to unveil the mechanisms underlying this relationship.

This review focuses on the interactions between the commensal microbiota and autoimmune liver diseases. We propose the importance of the microbiome-gut-liver triangle in autoimmune and cholestatic liver injury. Moreover, we will give an outlook on the diagnostic and therapeutic potential targeting this triangle.

## Method

A systematic search was performed for abstracts cited on PubMed, CINAHL, EMBASE, Cochrane Library, and Trip Pro up to November 2021. The only restriction imposed on the literature search was for English language abstracts only. After identifying relevant titles, the abstracts of these articles were surveyed to decide if the study contained material pertinent to the review. A manual cross-reference search of bibliographies was also performed to identify potentially relevant articles.

## Autoimmune and cholestatic liver diseases—definition and clinical perspective

### Autoimmune hepatitis

AIH is a chronic inflammatory disease with a female predominance that affects all age groups and ethnicities. The condition can lead to cirrhosis and liver failure with the subsequent need for liver transplantation or death [16]. The diagnosis of AIH is based on the presence of circulating non-organ specific autoantibodies, hypergammaglobulinemia with a selective elevation of serum IgG levels, compatible liver histology, and the exclusion of other causes, especially viral hepatitis and drug-induced liver injury [17]. Elevated aminotransferases are typical but may spontaneously normalize, despite histopathological evidence of persisting inflammatory disease [18]. Cirrhosis is present in one-third of adult patients at diagnosis [17]. Standard treatment includes corticosteroids and azathioprine, the only approved agents for AIH treatment [18]. The main treatment goals are normalization of IgG and transaminase levels (biochemical remission) and lack of inflammatory activity on liver histology (histological remission). Liver-related morbidity and mortality significantly increase among patients without a proper response to first-line treatment [1]. Several drugs are used off-label for second- or third-line treatment of patients with incomplete remission or intolerance to first-line therapy, including mycophenolate mofetil, calcineurin inhibitors, anti-TNF-antibodies, and B cell depleting monoclonal antibodies. Large multicenter trials on salvage therapies are urgently required [1, 17]. Since diagnostic tests lack specificity and sensitivity, new biomarkers for diagnosis and treatment response monitoring are warranted [1].

### Primary biliary cholangitis

PBC affects mainly women over the age of 40 years [1]. PBC should be suspected in patients with chronic cholestasis, especially with alkaline phosphatase elevation, after exclusion of other causes of liver disease [19]. The diagnosis can be made based on the basis of elevated alkaline phosphatase and the presence of antimitochondrial antibodies (AMAs) [20]. AMAs can be found in 95% of PBC patients, and the majority of AMA-negative PBC patients have specific antinuclear antibodies [19]. Diagnostic liver biopsy is, therefore, no longer required for the majority of patients [21].

UDCA (ursodeoxycholic acid) is recommended as the first-line treatment of PBC. Several criteria for treatment response, including liver biochemical values, have been suggested [19, 21]. Depending on the response criteria, 25–50% of patients suffer from UDCA treatment failure. Insufficient response after 1 year of treatment is associated with an increased risk of disease progression, including the development of hepatocellular carcinoma and end-stage liver disease [1]. For patients with insufficient response to UDCA, combination therapy with obeticholic acid is licensed second-line treatment, and those intolerant to UDCA can be treated with obeticholic acid as monotherapy [21]. Off-label treatment with bezafibrate in patients with inadequate response to UDCA alone has favorable therapeutic efficacy, including relief of itching [1, 19, 22]. Several novel agents are currently under investigation. The goal of current trials is to maximize response in insufficient responders to UDCA while maintaining an acceptable safety profile. New biomarkers to predict inadequate response to UDCA and stratify the risk of disease progression are needed [1].

### Primary sclerosing cholangitis

PSC is a rare chronic cholestatic liver disease characterized by inflammation, fibrosis, and obstruction of intrahepatic or extrahepatic bile ducts [4]. The pathogenesis of PSC remains elusive. The condition frequently reduces health-related quality of life and increases morbidity and mortality [1]. Chronic cholestatic liver test abnormalities, mainly alkaline phosphatase elevation, are typical for PSC but may be absent in early disease in up to 20% of patients [4]. The noninvasive magnetic resonance cholangiopancreatography is the preferred method to establish the diagnosis and typically shows multifocal strictures of intra- and extrahepatic bile ducts and prestenotic dilatations. Endoscopic retrograde cholangiopancreatography, associated with a risk for complications such as pancreatitis, is mainly reserved for a diagnostic sampling of new or progressing strictures or therapeutic interventions for mechanical bile duct obstruction [23]. Liver biopsy is recommended in patients with suspected PSC but normal cholangiography to diagnose small duct PSC. The pathognomonic histological sign is a concentric periductal “onion-skin” fibrosis, which, however, is seen infrequently [19].

Patients with PSC have a poor long-term prognosis, with up to 40% requiring liver transplantation and 20–30% developing cholangiocarcinoma [4]. About 70% of PSC patients suffer from associated inflammatory bowel disease and require surveillance colonoscopies due to the highly increased risk of colorectal cancer [4]. Liver transplantation is the only curative treatment option, but 20–40% of patients suffer from recurrence of PSC. There are no effective drugs to prevent disease progression, liver transplantation, cholangiocarcinoma, or death [1]. Ursodeoxycholic acid is widely used, but its use remains controversial. There was no clinical benefit for several immunosuppressive agents, antibiotics, and other drugs [1, 9, 20]. Thus, medications for PSC that improve survival and transplant-free survival are urgently needed. Furthermore, new biomarkers for diagnosis, outcome prediction, and monitoring of PSC are urgently required [1, 9].

## Microbiome-immunity crosstalk in health and disease

Virtually, all human body surfaces are colonized by complex communities of microorganisms (microbiome); these microbes are predominantly of bacterial origin, and the most densely populated human habitat is the intestine. Rapid developments in culture-independent molecular microecology methods have facilitated a rapidly growing body of research on the role of the human microbiome in health and disease [24, 25]. The mammalian immune system features an intricate network of innate and adaptive components. Both the innate and adaptive immune system elements are present in all tissues and synergistically protect the organism’s homeostasis from external threats and internal danger signals [26]. A disruption of the intestinal microbiome through interference with the environment (such as the use of antibiotics, diet, or geographical adjustments), impairment of the interfaces between host and microbiome, or changes in the immune system can lead to a systemic spread of commensal microorganism, susceptibility to infection, and aberrant immune responses [27]. Germ-free animals were indispensable to understanding the mechanisms underlying microbiome-immunity interactions. Microbial colonization of the mammalian host’s body surfaces is a milestone event profoundly shaping and educating the immune system. It is hypothesized that most events in the context of colonization and immune system development occur during a limited “window of opportunity” in the first years of life; this creates simultaneously a chance to establish immune homeostasis as well as a susceptibility towards perturbation and maladaptation with potentially long-term consequences for immunity-related health [28, 29]. Several modulators affect initial microbial colonization, including the mode of delivery [30]. In the past years, research on microbiome-immunity interaction has burgeoned. More comprehensive reviews on that subject matter can be found elsewhere (e.g., [27]). Herein, we give a concise overview we deem relevant to this review’s main subject.

### Microbiome and innate immunity

The innate immune system and the commensal microbiome are intricately linked. Antimicrobial peptides (AMPs) belong to the phylogenetically oldest components of innate immunity. Intestinal AMPs are mainly produced by Paneth cells and shape the microbiome’s configuration [31]. Pattern recognition receptors (PRRs), notably toll-like receptors (TLRs), are innate immune sensors responding to microbial signals. TLRs facilitate immune signaling by recruiting certain adapter elements (e.g., MyD88) and by activating transcription factors (e.g., NF‐κB); together, these processes result in inflammatory cytokine expression and type I interferon production [32]. Recent evidence shows that PRR ligands are derived from eternal pathogens and abundantly produced by commensal microbes [33]. The TLR expression in intestinal epithelia displays a high degree of spatial, cell-subtype-specific, and temporal diversity [34]. The role of TLR5 in shaping the intestinal microbiome has been studied comprehensively [35, 36]. TLRs are also abundantly expressed by various cells within the liver, including Kupffer cells, dendritic cells, hepatic stellate cells, endothelial cells, and hepatocytes [37]. Animal studies have shown that hepatic TLR4 signaling induced by the bacterial cell wall component lipopolysaccharide leads to liver inflammation and fibrosis [38]. Other PRRs proposed to shape the gut microbiome composition are NOD-like receptors (NLRs). Nucleotide-binding oligomerization domain-containing protein 1 (NOD1) serves as an innate sensor aiding adaptive lymphoid tissues and maintaining intestinal homeostasis [39]. Some NLRs assemble into cytosolic multiprotein complexes called inflammasomes; their pleiotropic immune functions are reviewed in detail elsewhere [40]. Inflammasomes activate caspases through cleavage. Activated caspases promote the maturation of the pro-inflammatory cytokines IL-1β and IL-18 and induce a lytic type of cell death termed pyroptosis [40]. An extensive array of microbial signals originating from pathogens and commensal bacteria can influence inflammation assembly and activation. Different inflammasomes have been implicated in the regulation of intestinal microbiome composition, notably the NLRP6 inflammasome [41]. Emerging research links inflammasome signaling within hepatocytes, macrophages, and Kupffer cells with inflammatory liver injury [42, 43]. The liver is host to a wealthy population of innate immune cells stimulated by commensals. Macrophages in the liver comprise subsets of different cell populations such as Kupffer cells, accounting for 80–90% of all resident macrophages in the body, and recruited monocyte-derived macrophages [44]. Besides macrophages, hepatic innate immune cells include natural killer cells, natural killer T cells, and γδ T cells [45]. Myeloid and lymphoid resident immune cells concentrate around periportal regions of the hepatic lobule. This asymmetric immune zonation results from sustained MYD88-dependent signaling induced by commensal bacteria in liver sinusoidal endothelial cells [46].

### Microbiome and adaptive immunity

Microbiota and adaptive immunity interlace in far-reaching bidirectional interaction. Recent research provides a detailed depiction of the crosstalk between the gut microbiome and CD4 + regulatory T cells. Notably, a subset of colonic regulatory CD4 + T cells lacks differentiation in GF mice resulting from the absence of bacterial consortia capable of fermenting dietary fiber into short-chain fatty acids (SCFAs) [31]. The CD4 + subset of Th17 cells is subject to intensive research due to its ambiguous roles in protective immune responses and chronic inflammation [32]. The intestine harbors different Th17 cell populations. The particular bacteria eliciting their differentiation define their inflammatory inclination. Th17 cells induced by SFB are non-inflammatory, while Th17 cells stimulated by *Citrobacter rodentium* are a source of pro-inflammatory cytokines [33]. Contemporary research has shed light on the impact of the microbiota on CD8 + T cell memory. The microbiota promotes CD8 + T cell long-term survival as memory cells guided by microbial metabolite-induced metabolic rewiring of activated CD8 + T cells [34]. A part of primary bile acids secreted into the intestine escape enterohepatic circulation into the colon. Here, they are converted by gut bacteria into bioactive secondary bile acids [35]. In the colon, secondary bile acids modulate a vital population of colonic FOXP3 + regulatory T (Treg) cells expressing the transcription factor RORγ, a population crucial for the host’s immunological homeostasis [36].

Some bile acid metabolites seem to affect adaptive immunity. Derivatives of lithocholic acid (LCA), 3-oxoLCA, and isoalloLCA, directly modulate T helper cells. While 3-oxoLCA inhibits Th17 cell differentiation, isoalloLCA seems to enhance Treg differentiation. In mice, administration of 3-oxoLCA and isoalloLCA reduced Th17 and increased Treg cell differentiation in the intestinal lamina propria [37]. Interestingly, Odoribacteraceae strains seem to produce isoalloLCA, which has been suggested to inhibit Gram-positive pathogen expansion in vivo [38].

## Intestinal barrier function in liver diseases

The intestinal epithelium is the principal barrier to preserve intestinal compartmentalization and safeguard the host from enteric bacteria. Intestinal barrier dysfunction contributes to diseases affecting the liver and other internal organs [39]. The intestinal barrier incorporates physical, immunological, and microbial elements. The physical barrier consists of epithelial and mucus components. The intestinal epithelial layer’s integrity is stabilized by occlusive intercellular molecular joints termed “tight junctions” (TJs) [40]. Commensal microbes reinforce the gut barrier through various mechanisms [41]. Under normal physiological circumstances, intestinal goblet cells continuously produce mucins to replenish the mucus layer. Commensals stimulate microbe recognition receptors (such as TLRs) in intestinal cells, thereby triggering mucins and AMPs [42]. Many commensal bacteria produce short-chain fatty acids, such as butyrate, from the fermentation of insoluble fiber. Among butyrate’s many functions, TJ barrier maintenance has recently been described [43]. In the intestinal barrier’s failure, even bacteria beneficial under normal physiological conditions can kindle inflammation and elicit organ injury [44]. Increased gut permeability leads to an influx of microbe-associated molecular patterns (MAMPs; also termed pathogen-associated molecular patterns [PAMPs]) into the systemic circulation, stimulating an immune response. PAMPs originating from the gut, such as lipopolysaccharide or microbial RNAs, can reach the liver via the portal circulation and induce liver inflammation and fibrosis, e.g., mediated by TLR4 [45–47]. Various mechanisms are causing a leaky intestine; they are still incompletely understood and subject to vibrant research. Such mechanisms include physical trauma and toxins such as alcohol, TJ severance, altered epithelial stem cell turnover, and alterations to the mucus layer texture [48, 49]. Emerging research points towards a close relationship between gut microbiome alterations, intestinal leakiness, and autoimmunity [50]. The molecular mechanisms underlying this connection are mostly unclear. Tools to identify molecular agents of microbial origin impacting the intestinal barrier are emerging [51].

## Evidence from epidemiological and clinical studies

Infections are thought to play a significant role in the development and exacerbation of autoimmune diseases. Autoimmunity following infection can result from several mechanisms, including molecular mimicry [52]. Recent epidemiological data suggest that antibiotic intake during childhood can lead to gut dysbiosis and seems to be associated with autoimmune disorders in adulthood [53].

### Autoimmune hepatitis

Environmental factors including antibiotics, alcohol consumption, and diet have a profound impact on the intestinal microbiome. Microbiota communities resulting from specific environmental modifiers such as antibiotics might increase the susceptibility to develop AIH [54]. In a study including 72 AIH patients and 144 healthy controls, exposure to antibiotics within 12 months before AIH diagnosis was an independent risk factor for AIH manifestation [55].

Up to 20% of AIH patients present specific autoantibodies to soluble liver antigen/liver-pancreas (SLA/LP) protein. Structural similarity between a region of the surface antigen PS 120 from *Rickettsia* spp. and immunodominant regions of SLA/LP autoepitope has been detected in an in silico study, supporting the hypothesis that molecular mimicry might trigger AIH [56]. Future studies should assess the frequency of memory B and T cells specific to PS 120 protein epitopes in AIH patients and healthy controls to conclude that the suggested peptides are authentic immunodominant T cell epitopes. Furthermore, the potential association of infections with other bacteria and AIH development should be investigated in future studies [57].

### Primary biliary cholangitis

Several genetic and environmental factors are assumed to play a role in the onset and perpetuation of bile duct injury in PBC, including infectious and chemical exposures leading to molecular mimicry or modification of autoantigens [58]. Bacterial infections seem to constitute a pivotal environmental risk factor for PBC, especially in female patients [59]. Molecular mimicry and immunological cross-reactivity between several bacteria and human mitochondrial antigens have been suggested to contribute to the pathogenesis of PBC. The disease-specific antimitochondrial autoantibodies are directed against members of the 2-oxo-acid dehydrogenase complex family of enzymes. The pyruvate dehydrogenase complex E2 subunit (PDC-E2) represents a significant autoantigen, and more than 95% of patients with PBC show serologic immune responses to PDC-E2 [60].

**Fig. 1:**
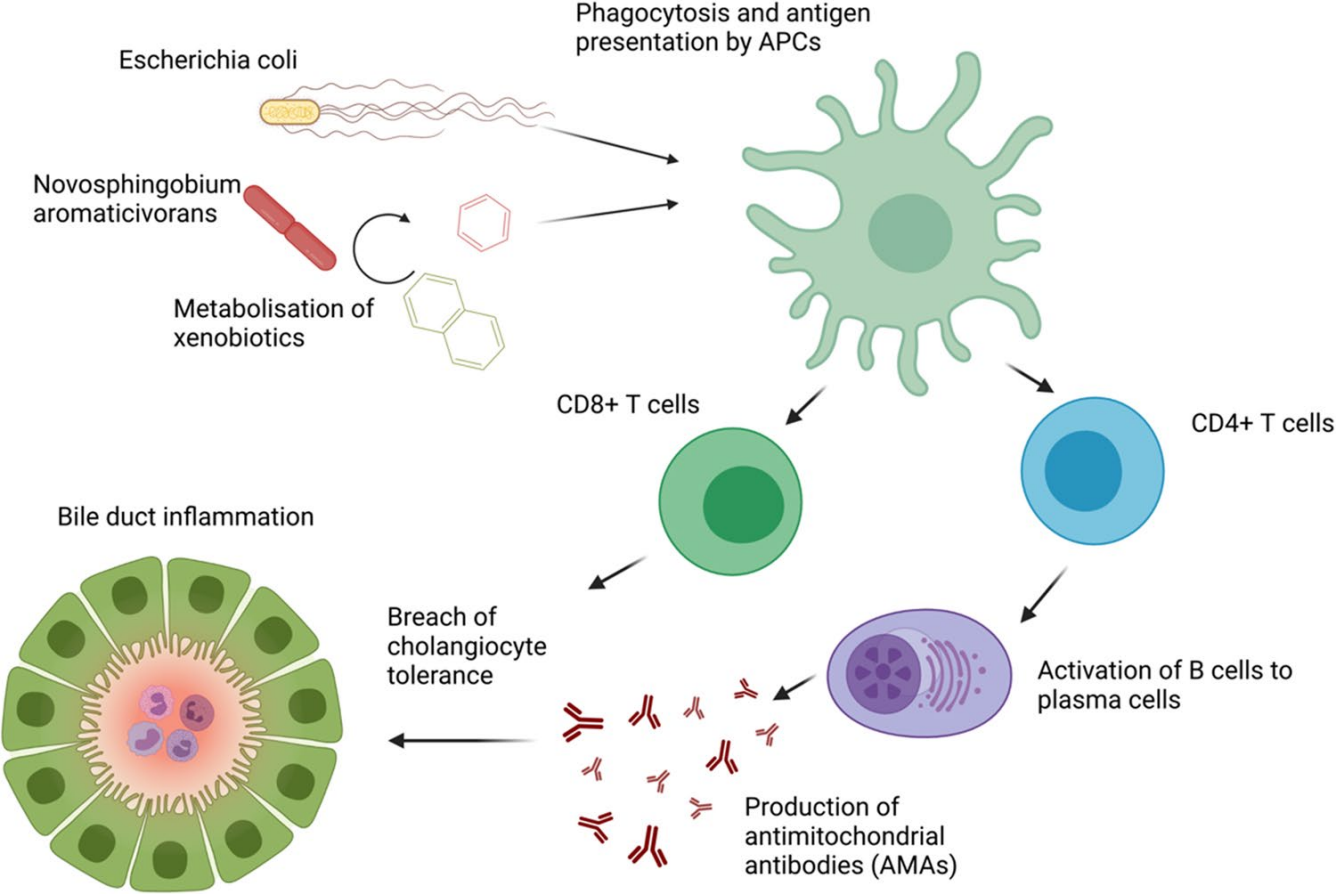
Potential induction of PBC by *E. coli* or *N. aromaticivorans* by molecular mimicry. *E. coli* or *N. aromaticivorans* are recognized and phagocytized by antigen-presenting cells (APCs). APCs present bacterial antigens with structural similarity to PDC-E2 to T cells by MHC-I and MHC-II. Autoreactive CD4 + T cells induce the production of antimitochondrial antibodies (AMAs), which with autoreactive CD8 + T cells lead to a breach of cholangiocyte tolerance and nonpurulent bile duct destruction

Several large-scale, case–control studies have observed a significantly higher prevalence of recurrent urinary tract infections in patients with PBC, whereby *Escherichia coli* was identified as the predominant pathogen. Molecular mimicry between the human and *E. coli* PDC-E2 have been demonstrated, and infection with *E. coli* seems to result in the production of the disease-specific antimitochondrial autoantibodies [59] (Fig. 1). A case–control study concluded that the ubiquitous bacterium *Novosphingobium aromaticivorans* is another candidate that might be involved in the pathogenesis of PBC. Two bacterial proteins show a high degree of homology with the dominant immunogenic domain of the PDC-E2, representing the highest level of homology between this mitochondrial autoantigen and any known microorganism. Sera from 77 out of 77 PBC patients (100%) reacted against the investigated bacterial proteins, whereby the reactivity was at least 100-fold higher than the reactivity against *E. coli*. None of the 195 control sera responded against *Novosphingobium aromaticivorans*. The authors suggest that *N. aromaticivorans* might break tolerance to self PDC-E2 by two independent mechanisms, including alteration of bacterial PDC-E2 or host PDC-E2 by xenobiotics metabolism [61]. Furthermore, a potential impact of mycobacteria, chlamydia, helicobacter species, and lactobacilli on PBC pathogenesis has been suggested [60].

Another study demonstrated significant alterations of circulating bile acids in treatment-naïve PBC patients that were strongly associated with disease progression. PBC patients showed gut dysbiosis that correlated with the bile acid profile compared to healthy controls. UDCA treatment reversed the bile acid profile and dysbiosis in PBC patients. Thus, bile acid profiling could contribute to PBC patients’ diagnosis and disease status assessment. Altering gut microbiota might allow modulation of the bile acid profile and, subsequently, be harnessed for PBC patients’ treatment [62].

### Primary sclerosing cholangitis

The intestinal and biliary microbiome are increasingly thought to play a pathogenetic role in PSC. The involvement of the gut microbiota in the pathogenesis is supported by the fact that bacterial RNA was found in liver tissues, and bacteria and fungi were detected in most bile duct cultures of PSC patients. Moreover, an increased T cell response to microbial antigens was observed in PSC patients independent of IBD presence [63]. In another study, the persistence of biliary candidiasis was associated with markedly reduced transplantation-free survival in PSC patients [64].

It is debated whether antibiotic treatment might benefit patients with PSC with selective positive effects reported for metronidazole and vancomycin, specifically [65–68]. These potential positive effects might be explained either via direct effects on the microbiome or via host-mediated mechanisms. A recent meta-analysis concluded that vancomycin might be the most promising antibacterial pharmacotherapy. However, the ideal antibiotic drug, dose, regimen, and potential long-term detrimental consequences remain largely unknown [69].

Tacrolimus, a macrolide with immunosuppressive activity produced by the soil bacterium *Streptomyces tsukubaensis*, significantly decreased several biochemical markers, including alkaline phosphatase, after about 1 year of treatment. However, no effect on ERCP or liver histology was observed [70]. A randomized placebo-controlled study including 80 PSC patients investigated the disease progression in patients with UDCA and metronidazole compared to UDCA and placebo. In the metronidazole group, serum alkaline phosphatase levels and the Mayo Risk Score significantly improved after 36 months. However, on liver histology and ERCP, no significant impact on progression was found [66]. Treatment with vancomycin improved clinical symptoms and biochemical markers such as alanine aminotransferase in 14 children with PSC, especially in those without cirrhosis [68]. A study including 17 pediatric patients with ulcerative colitis and PSC or autoimmune sclerosing cholangitis reported clinical, biomarker, mucosal, and histological remission of colitis following vancomycin [71]. However, a recent study including 264 pediatric PSC patients showed no improvement in outcome after 1 year of vancomycin or UDCA compared to observation without treatment [72].

In a randomized, double-blind study, 35 adult PSC patients received metronidazole or vancomycin in two different doses each for 12 weeks. A significant decrease in alkaline phosphatase was observed after 12 weeks of both high- and low-dose vancomycin. The Mayo Risk Score was lower at the end of treatment in the low-dose metronidazole and low-dose vancomycin group compared to the respective high-dose groups [65]. Another study including minocycline for 1 year observed significantly improved serum alkaline phosphatase activity and Mayo Risk Score. Liver histology was not assessed [67].

Longer-term placebo-controlled randomized trials on the safety and efficacy of antibiotic treatment with more definite primary endpoints such as transplant-free survival or validated surrogate biomarkers in PSC patients are needed [69].

PSC is often associated with atypical perinuclear antineutrophil cytoplasmic antibodies (p-ANCAs) directed against human beta-tubulin isotype 5 (TBB-5). These antibodies cross-react with FtsZ, the evolutionary bacterial precursor protein of TBB-5, suggesting an abnormal immune response to intestinal microbiota [73].

Patients with PSC compared to healthy controls have increased Th17 responses towards pathogen stimulation in vitro, independent of the presence of IBD. Within the portal tracts of PSC livers, IL-17A-expressing lymphocytes and bacterial RNA were found [63]. A recent study observed that PSC patients show significantly increased IL-17A-producing peripheral blood CD4 + T cells than healthy controls, indicating increased Th17 differentiation in vitro. Monocytes from PSC patients were shown to produce more IL-1β and IL-6, cytokines known to drive Th17 cell differentiation, following stimulation with *Candida albicans* and *Enterococcus faecalis*. In primary human cholangiocytes, monocytes activated by microbes induced the secretion of Th17 and monocyte-recruiting chemokines chemokine (C–C motif) ligand (CCL)-20 and CCL-2. Since monocytes may provide the functional link between microbiota and T cells, their potential as a therapeutic target in PSC should be studied in the future [74].

Trivedi and colleagues have demonstrated that circulating vascular adhesion protein (VAP)-1 levels are increased in PSC patients [75]. This enzyme facilitates the adhesion of intestine-derived lymphocytes to hepatic endothelial cells; circulating VAP-1 levels were correlated with adverse outcomes in PSC patients. The most crucial enzymatic substrate promoting these effects was the aminothiol compound cysteamine; this molecule is secreted by inflamed colonic epithelium and gut bacteria [75].

Autoantibodies against distinct splicing variants of glycoprotein 2 (GP2), an intestinal receptor of the bacterial adhesin FimH, frequently occur in a subgroup of PSC patients [76]. These patients display a more severe phenotype and poorer survival due to cholangiocarcinoma development. Anti-GP2 IgA directly links prognosis in PSC with bacterial factors and may serve as a clinically valuable tool for risk stratification in PSC [77].

Fecal microbiota transplantation (FMT) may alter the host microbiome. A pilot clinical trial examined the efficacy of FMT in PSC. This study was small and enrolled only ten patients, of whom only six underwent FMT. Three patients showed a significant (50% or more) decrease in serum alkaline phosphatase by week 26. Microbiome diversity increased in all PSC patients receiving FMT 1 week after the start of FMT. This increase remained stable in most participants [78]. This preliminary trial allows the conclusion that FMT is safe in PSC and shows potential for clinical improvement. However, the limited number of patients and time frame do not qualify for further findings or recommendations.

### Evidence linking AILDs with viral infections

Although both conventional microbiological and next-generation sequencing–based studies (see below) emphasized the triggering role of bacteria in AILDs, substantial evidence suggests that viral infections may elicit the manifestation of AILDs in vulnerable individuals as well [79].

In AIH, the hepatitis C virus (HCV) has been discussed as a candidate trigger because the virus shares high amino acid sequence homology with the auto-antigenic target of anti-LKM-1 autoantibodies cytochrome P4502D6 (CYP2D6) [80]. Other potential viral triggers of AIH encompass hepatitis B virus, hepatitis E virus, cytomegalovirus, and herpes simplex virus [81].

In a collaborative effort, Xu and colleagues found evidence for the presence of human betaretrovirus (also termed human-mouse mammary tumor virus) in biliary epithelia in the majority of explanted PBC patients’ livers [82]. Moreover, biliary epithelial cells developed phenotypic signs of PBC when cultivated with supernatants containing the human betaretrovirus or the structurally related murine mammary tumor virus [82]. However, another group could not replicate this finding [83]. Another study reported that the detection of this virus in diseased human livers is non-specific [84]. Thus, firm evidence for a viral infection underlying PBC is lacking.

Anecdotal evidence from case reports linked PSC with HCV infection and human T-lymphotropic virus type 1 [85–87]. However, to the best of our knowledge, no cohort or mechanistic studies confirm these associations.

In light of the recent COVID-19 pandemic, reports emerged associating AIH and AIH/PBC variant syndrome with severe acute respiratory syndrome coronavirus 2 (SARS-CoV-2) infection and vaccination against SARS-CoV-2 [88–90]. However, additional evidence is warranted to substantiate this potential link.

To summarize, various studies have linked AIH, PBC, and PSC to viral triggers. However, so far, none of these studies has consistently and unequivocally satisfied the Henle-Koch postulates to prove viral pathogenesis in AILDs. The viral infection hypothesis in the pathogenesis of AILDs merits further research.

## Evidence from human case–control microbiome studies

Next-generation sequencing’s availability and affordability have reinvigorated the interest in studying the role of commensal microorganisms in autoimmune liver diseases. The past 5 years saw a surge of human case–control microbiota studies in this field. While interpreting microbiome studies in clinical populations, it is paramount to acknowledge the variation and biases introduced by different experimental protocols and the multiple sources of confounding in human cohorts [91]. It is furthermore critical to understand that microbial communities differ substantially between the gut lumen and mucosal surfaces. At the same time, mucosal surfaces of different segments of the digestive tract vary considerably as well. Differing sampling methods, such as biopsies, brushing, or luminal washes, may explain the higher variability of results observed in mucosal studies than luminal studies.

### Autoimmune hepatitis

The microbiome may serve as a reservoir of antigens initiating or maintaining autoimmune responses in AIH [57]. Several studies have investigated the microbiome configuration in patients with AIH compared to either healthy or diseased controls (Table 1). All studies published so far were cross-sectional and relied on sequencing the 16S gene to the best of our knowledge.

**Table 1:** Overview of taxonomic alterations in case–control studies on the gut microbiota in AIH

Reference	Study population	Biological source	Sequencing technique	Enriched in AIH	Depleted in AIH
Lou et al., 2020 [92]	37 AIH; 78 HC	Feces	16S, V3–V5	34 OTUs including *Enterobacteriaceae*, *Veillonella*, *Ruminococcaceae*_uncultured, *Roseburia*, and *Bacteroides*	5 OTUs including *Bacteroides*, *Bilophila*, *Blautia*, and *Lachnospiraceae*_uncultured
Elsherbiny et al., 2020 [93]	15 AIH; 10 HC	Feces	16S, V3–V4	*Faecalibacterium*, *Blautia*, *Streptococcus*, *Bacteroides*, *Haemophilus*	*Prevotella*, *Parabacteroides*, *Dilaster*
Liwinski et al., 2020 [94]	72 AIH; 99 PBC; 81 UC; 95 HC	Feces	16S, V1–V2	vs HC: *Streptococcus*, *Lactobacillus*, *Veillonella* vs PBC: *Faecalibacterium*, *Haemophilus*, *Ruminococcaceae* unclassified vs UC: UBA 1819, *Phascolarctobacterium*, various *Ruminococcaceae* genera, *Odoribacter*, *Senegalimassilia*, *Subdoligranulum*, *Coprobacter*, *Lachnospiraceae* NK4A136, *Parabacteroides*, *Butyricimonas*	vs HC: *Lachnospiraceae* ND3007, *Intestinibacter*, *Erysipelotrichaceae*, *Bifidobacterium*, *Lachnispiraceae* FCS020, *Clostridium* family XIII AD3011, *Faecalibacterium* vs PBC: *Bifidobacterium*, *Sellimonas*, UBA89, *Blautia*, *Romboutsia*, *Coprococcus*, *Lachnoslostridium*, *Flavonifractor*, DTU089, *Butyricicoccus* vs UC: *Bifidobacterium*, *Blautia*, *Erysipeloclostridium*, *Intestinibacter*, *Phascolarctobacterium*
Wei et al., 2020 [95]	91 AIH; 98 HC	Feces	16S, V3–V4	*Lactobacillus*, *Klebsiella*, *Veillonella*	*Ruminococcaceae* genus (unknown), *Rikenellaceae* genus (unknown), *Oscillospira*, *Parabacteroides*, *Coprococcus*

*AIH*, autoimmune hepatitis; *HC*, healthy controls; *PBC*, primary biliary cholangitis; *UC*, ulcerative colitis.

In a preliminary study involving 24 Chinese patients with autoimmune hepatitis and eight healthy volunteers, Lin et al. have identified an increased intestinal permeability by decreasing tight junction integrity and increased serum levels of bacterial lipopolysaccharide (LPS) [96]. In a large Chinese case–control study, Wei et al. have studied the gut microbiome in steroid-naïve AIH patients [95]. Compared to healthy controls, AIH patients displayed a reduced microbiome diversity and a distinct overall microbiome composition featuring a depletion of certain obligate anaerobes (such as *Faecalibacterium*) and an expansion of the genus *Veillonella*. Notably, the species *Veillonella dispar* correlated with serum aspartate aminotransferase and liver inflammation [95]. In a cross-sectional study from Germany involving AIH patients with immunosuppressive treatment, Liwinski et al. have in part confirmed critical findings of the study by Wei et al., such as altered overall microbiota composition, reduced biodiversity, reduced relative abundance of beneficial anaerobic species (such as *Faecalibacterium prausnitzii*), and an expansion of *Veillonella* [94].

Moreover, a relative increase in the facultative anaerobic genera *Streptococcus* and *Lactobacillus* were detected. A unique finding of this study was a pronounced depletion of genus *Bifidobacterium*, which had a solid association with failure to achieve remission of liver inflammation. Importantly, this study has shown that gut microbial alterations in AIH are disease-specific and that AIH and PBC can be distinguished well based on the microbiota profile [94].

Another Chinese study by Ren and colleagues confirmed the downward trend of fecal microbial diversity in patients compared to healthy controls [92]. While the critical finding of *Veillonella*’s enriched fecal relative abundance was confirmed, a key contradiction to the former studies was an increased relative abundance of *Faecalibacterium* in AIH patients [92].

The oral microbiome is an emerging frontier in microbiota research. One study found a significantly higher frequency of genus *Veillonella* and a lower frequency of genus *Streptococcus* and genus *Fusobacterium* in the oral cavity of AIH patients compared to healthy controls [97].

Although converging evidence from the studies summarized above is encouraging, the disparate and sometimes contradictory results warrant direct comparisons of larger international cohorts to validate disease‐specific microbial signatures in AIH independent of varying dietary and geodemographic circumstances. Evidence from metagenomic shotgun sequencing and functional characterization using functional metagenomic profiling, metatranscriptomics, proteomics, or metabolomics is still lacking in AIH patients. It may unveil a picture more complex than the one gained by way of 16S gene sequencing. Likewise, no analysis of viral or fungal communities has been published to date. Moreover, profiling the intestinal mucosal microbiome is still lacking and may complete the account of intestinal microbial alterations associated with AIH.

### Primary biliary cholangitis

Li and colleagues have characterized the gut microbiome using 16S sequencing in a Chinese cohort of patients with early-stage PBC. Most patients received UDCA [98]. The gut microbiome of PBC patients was depleted of some potentially beneficial bacteria, such as *Ruminococcus bromii*. On the other hand, taxa such as phylum Proteobacteria, family Enterobacteriaceae, and the genera *Veillonella*, *Streptococcus*, and *Klebsiella*, potentially entailing pathogens, were enriched [98]. Tang et al. recruited a large Chinese cohort of treatment-naïve PBC patients and healthy controls. A prospective trial was conducted in a subgroup of patients with PBC with microbiota-profiling before and after 6 months of treatment with UDCA [99]. A significant reduction of within-individual microbial diversity was observed in untreated PBC patients. Potential pathogens such as *Klebsiella*, *Haemophilus*, *Streptococcus*, and *Veillonella* were enriched in UDCA-naïve PBC patients compared to controls. The altered abundance of six PBC-associated genera was restored after 6 months of treatment with UDCA. In particular, *Faecalibacterium*, enriched in controls, was decreased in gp210-positive compared to gp210-negative patients [99]. In a recent cross-sectional study including 23 PBC patients, fecal microbiota and metabolic profiles were investigated. Fecal acetate and SCFAs were found higher in PBC patients with advanced fibrosis [100]. Furukawa et al. have studied the relationships between clinical profiles, biochemical response to UDCA, and gut microbiome composition in a cohort of Japanese patients with PBC treated for at least 1 year with UDCA [101]. Altered gut microbial composition with loss of *Clostridiales* commensals was observed in patients with PBC. Among UDCA non-responders, *Faecalibacterium* (a butyrate-producing and potentially beneficial taxon) showed confounder-robust significantly lower relative abundance. The authors conclude that a decrease in *Faecalibacterium* abundance might predict the prognosis of patients with PBC [101]. Table 2 summarizes the findings from NGS case–control studies in PBC.

**Table 2:** Overview of taxonomic alterations in case–control studies on the gut microbiota in PBC

Reference	Study population	Biological source	Sequencing technique	Enriched in PBC	Depleted in PBC
Chen et al., 2020* [62]	65 PBC; 109 HC	Feces	16S, V3–V4	*Enterobacteriaceae* genus (unknown), *Prevotella*, *Veillonella*, *Fusobacterium*, *Haemophilus*, *Streptococcus*, *Clostridiaceae* genus (unknown), *Pseudomonas*, *Citrobacter*, *Lactobacillus*, *Salmonella*, *Clostridium*, *Klebsiella*, *Sneathia*	*Mogibacteriaceae* genus (unknown), *Blautia*, *Christensenellaceae* genus (unknown), *Butyricimonas*, *Akkermansia*, *Odoribacter*, *Dialister*, *Rikenellaceae* genus (unknown), *Oscillospira*, *Faecalibacterium*, *Phascolarctobacterium*, *Sutterella*, *Clostridiales* genus (unknown), *Barnesiellaceae* genus (unknown), *Bacteroides*
Tang et al., 2018* [99]	60 PBC; 80 HC	Feces	16S, V3–V4	*Klebsiella*, *Lactobacillus*, *Clostridium*, *Pseudomonas*, *Haemophilus*, *Streptococcus*, *Veillonella*, *Enterobacteriaceae* genus (unknown)	*Oscillospira*, *Faecalibacterium*, *Sutterella*, *Bacteroides*

*Cohorts overlap partially.

*HC*, healthy controls; *PBC*, primary biliary cholangitis.

To conclude, the gut microbiome is altered in PBC patients and may be critical for the onset, progression, and prognosis by interacting with metabolism and immunity. Attempts towards prospective study designs and integrative analyses utilizing several data domains shed new light on the pathophysiology of PBC. However, studies relying on metagenomic shotgun sequencing and large-scale multi-omic integration are still lacking. No analysis of commensal viral or fungal communities has been published so far. Also, we are not aware of any studies investigating the intestinal mucosal or biliary microbiome in PBC, which may yield interesting results aiding the findings from fecal profiling.

### Primary sclerosing cholangitis

There has been a bustle of studies in the past 5 years to characterize the gut microbiota in PSC/PSC-IBD compared to healthy controls (HCs) and IBD patients [102–111]. These studies covered the fecal and mucosal microbiome and reinforced the importance of the intestinal microbiome in PSC. Most reports focused on the bacterial microbiome. We have summarized the bacterial alterations unveiled by those publications in adult PSC patients in Table 3.

**Table 3:** Overview of taxonomic alterations in case–control studies on the gut microbiota in PSC

Reference	Study population	Biological source	Sequencing technique	Enriched in PSC	Depleted in PSC
Sabino et al., 2016 [111]	18 PSC only; 27 PSC-UC; 21 PSC-CD; 13 UC; 30 CD; 66 HC	Feces	16S, V4	vs HC: *Bacteroidetes*, *Fusobacteria*, *Streptococcus*, *Enterococcus*, *Lactobacillus*, *Fusobacterium*, *Veillonella* PSC-IBD vs PSC only: none	vs HC: *Firmicutes Anaerostipes* PSC-IBD. vs PSC only: none
Bajer et al., 2017 [110]	32 PSC-IBD; 11 PSC only; 32 UC 31 HC	Feces	16S, V4	vs HC: *Rothia*, *Enterococcus*, *Streptococcus*, *Clostridium*, *Veillonella*, *Haemophilus* (PSC only, and PSC-IBD); *Staphylococcus*, *Coprobacillus*, *Escherichia*, *Corynebacterium*, *Lactobacillus* (PSC-IBD) vs UC: *Rothia*, *Streptococcus*, *Veillonella*, *Blautia*; *Akkermansia muciniphila*, *Clostridium colinum*	vs HC: *Coprococcus* (C. catus), unidentified *Lachnospiraceae* genera, *Faecalibacterium prausnitzii*, *Ruminococcus gnavus*, *Prevotella copri* (PSC only, and PSC-IBD) *Phascolarctobacterium* (PSC-IBD); *Adlercreutzia equolifaciens* (PSC only) vs UC: *Fusobacteriaceae*
Kummen et al., 2017 [109]	44 PSC-UC; 11 PSC-CD; 30 PSC only; 36 UC; 263 HC	Feces	16S, V3–V4	vs HC: *Veillonella* (*V. dispar*, *V. parvula*) vs UC: *Veillonella* (*V. dispar*, *V. parvula*); *Akkermansia*, *Clostridium*, Ruminococcaceae	vs HC: ML615J-28 unknown genus, *Succinivibrio*, *Desulfovibrio*, RF32 unknown genus, *Phascolarctobacterium*, *Coprococcus*, *Lachnospiraceae* unknown genus, *Christensenellaceae* unknown genus, *Clostridiales* unknown genus, YS2 unknown genus, S24.7 unknown genus vs UC: *Dorea, Oscillospira*, *Citrobacter*
Rühlemann et al., 2017 [108]	38 PSC-UC; 35 PSC only; 88 UC; 98 HC	Feces	16S, V1–V2	vs HC: *Veillonella* (no difference observed compared to UC)	Not investigated
Rühlemann et al., 2019 [107]	75 PSC-IBD; 62 PSC only; 118 UC; 133 HC	Feces	16S, V1–V2	vs HC: *Veillonella*, *Streptococcus*, *Lactobacillus*, *Enterococcus*; *Proteobacteria* (*Gammaproteobacteria*), *Lactobacillales* (*Bacilli*), *Parabacteroides*, *Bacteroides* spp. vs UC: *Firmicutes* PSC-IBD vs PSC only: none	vs HC: *Coprococcus*, *Holdemanella*, *Desulfovibrio*, *Faecalibacterium*, *Clostridium* IV PSC-IB.D vs PSC only: *Bilophila* and *Bacteroides* OUT
Lemoinne et al., 2019 [112]	16 PSC-UC/IBD-U; 11 PSC-CD; 22 PSC only; 33 IBD; 30 HC	Feces	16S, V3–V4	vs HC: *Veillonella*, *Sphingomonadaceae*, *Alphaproteobacteria*, *Rhizobiales*	vs HC: *Ruminococcus*, *Faecalibacterium*, *Lachnoclostridium*, *Blautia*
Liu et al., 2021 [113]	37 PSC; 34 IgG4 cholangiopathies; 64 HC	Feces	16S, V3–V.4	vs IgG4/HC: *Turicibacter*, *Ruminococcus gnavus* group	vs IgG4/HC: *Eubacterium sineum* group, *Oscillospirales*, *Catenibacterium*, *Ruminococcaceae* UBA 1819 *Oscillospiraceae* UCG-005, *Lachnospiraceae* UCG-010, *Eubacterium ruminantium* group, *Eubacterium eligens* group, *Agathobacter*
Kummen et al., 2021 [114]	136 PSC; 93 IBD; 158 HC	Feces	Shotgun metagenomics	vs HC (meta-analysis; abundance): *Clostridium asparagiforme*, *Escherichia* unclassified vs HC (meta-analysis; prevalence): *Clostridium clostridioforme*, Clostridiales bacterium 1 7 47FAA, *Clostridium bolteae*, *Bifidobacterium bifidum*, *Clostridium symbiosum*, *Escherichia* unclassified, *Eggerthella* unclassified, *Eggerthella lenta*, *Clostridium citroniae*	vs HC (meta-analysis; abundance): *Coprococcus catus*, *Roseburia inulinivorans, Ruminococcus obeum*, *Subdoligranulum* unclassified, *Eubacterium rectale*, *Eubacterium siraeum*, *Bacteroides bacterium* ph8, *Barnesiella intestinihominis*, *Alistipesshahii*, *Bacteroides intestinalis* vs HC (meta-analysis; prevalence): *Coprobacter fastidiosus*, *Alistipes senegalensis*, *Eubacterium ramulus*, *Eubacterium hallii*, *Lachnospiraceae bacterium* 7 1 58FAA
Rossen et al., 2015 [106]	8 PSC-UC; 4 PSC-CD; 11 UC; 9 HC	Mucosal biopsy, ileocecum	16S (HITChip)	None	vs HC: Uncultured *Clostridiales* II vs UC: Uncultured *Clostridiales* II
Kevans et al., 2016 [105]	31 PSC-UC; 56 UC; 0 HC	Mucosal biopsy, left colon	16S, V4	None	None
Torres et al., 2016 [104]	13 PSC-UC; 6 PSC-CD; 1 PSC only; 13 UC; 2 CD; 9 HC	Mucosal biopsy, terminal ileum, right, and left colon	16S, V3–V4	vs IBD: *Barnesiellaceae*, *Blautia*, *Ruminococcus obeum*	None
Quraishi et al., 2017 [103]	11 PSC-IBD 10 IBD 9 HC	Mucosal biopsy, ascending, transverse, descending colon	16S, V3–V4	vs HC: *Escherichia*, *Lachnospiraceae*, *Megasphera*	vs HC: *Prevotella*, *Roseburia*, *Bacteroides*
Quraishi et al., 2020 [102]	10 PSC-IBD 10 UC; 10 HC	Mucosal biopsy, sigmoid colon	16S, V4	vs HC: *Bacilli*, *Pseudomonas*, *Streptococcus*, *Haemophilus parainfluenzae* vs UC: 24 taxa, including *Bacilli*, *Staphylococcus, Parvimonas* sp., *Bacteroides fragilis*, *Roseburia* spp., *Shewanella* spp., *Clostridium ramosum*, *Sphingomonas* sp., *Actinomyces*, *Rothia*	vs HC: *Lachnospiraceae* vs UC: 26 taxa, including *Lentisphaerae, Gammaproteobacteria*, *Enterobacteriaceae, Prevotellaceae*, *Paraprevotellaceae*, *Coriobacteriaceae*, *Erysipelotrichaceae*, *Desulfovibrionaceae*, *Myxococcales*, *Streptococcus*, *Blautia*

*CD*, Crohn’s disease; *HC*, healthy control; *IBD*, inflammatory bowel disease; *IBD-U*, inflammatory bowel disease—unclassified; *IgG4*, IgG4-associated cholangiopathy; *PSC*, primary sclerosing cholangitis; *UC*, ulcerative colitis.

Studies on the fecal microbiome in PSC populations have revealed several consistent themes. One such common motif is altered beta diversity (variation in taxonomic composition between samples) and decreased average alpha diversity (within-sample taxonomic diversity) in PSC compared to healthy controls. Compared with IBD populations, PSC is characterized by a specific kind of dysbiosis, although the differences tend to be less pronounced than between PSC patients and healthy subjects. The difference between PSC only and PSC-IBD appears to be marginal, indicating that liver pathology is the principal corollary of microbial dysbiosis. Regarding considerable heterogeneity in geography, study protocols, and clinical patient characteristics, it is not surprising that studies are not entirely consistent regarding the particular taxa altered in PSC (Table 3).

Nevertheless, a few taxa crop-up remarkably consistently as changed in PSC compared to healthy controls. In particular, the genus *Veillonella* is enriched in the stool of PSC patients in all studies scrutinized. Other genera frequently increased in relative abundance in PSC samples are *Enterococcus*, *Streptococcus*, and *Lactobacillus*. Moreover, short-chain fatty acids-producing anaerobes such as *Faecalibacterium* and *Coprococcus* were often found depleted in PSC patients. One study on a Japanese cohort with pediatric PSC reported alterations similar to adult patients, as shown in the studies summarized in Table 3 [115].

One is thus tempted to assume that *Veillonella* directly contributes to PSC’s pathophysiology. However, *Veillonella*’s increased abundance is not specific to PSC but also appears in cirrhosis of various origins, AIH, PBC [95, 99, 116], and non-hepatic disorders such as treatment-naïve Crohn’s disease [117]. Moreover, there is still no evidence for a causal role of *Veillonella* in liver injury.

A recent study by Kummen and colleagues applied metagenomic shotgun sequencing in a German and Norwegian cohort [114]. This study proved a decreased richness of microbial genes, increased prevalence of *Clostridium* species, and depletion of, e.g., *Eubacterium* species and *Ruminococcus obeum* in PSC. Patients with PSC displayed significant differences in the abundance of genes related to vitamin B_6_ synthesis and branched-chain amino acid synthesis. *Veillonella* was less prevalent than in previous 16S-based studies, but the authors still observed an increased prevalence of several Veillonella species in patients with PSC [114]. This study highlights the gain of transitioning to metagenomic shotgun sequencing and integrating corresponding blood and stool samples.

While bacteria were heretofore the research focus, investigators have now turned their attention to the gut’s fungal communities (mycobiome). Lemoinne et al. found that patients with PSC have a fungal gut dysbiosis characterized by a relative increase in biodiversity and an altered community composition. Moreover, they observed an increased relative abundance of *Exophiala* and a decreased abundance of *Saccharomyces cerevisiae* [112]. Bang and colleagues have found that the gut mycobiome of primary sclerosing cholangitis patients features an increase of *Trichocladium griseum* and *Candida* species [118]. In a prospective non-randomized trial, *Candida* was detected in the bile of 7 out of 49 (14%) PSC patients with dominant stenosis, one out of 18 PSC patients without dominant stenosis, and none of the patients without PSC. Biliary *Candida* was associated with more severe cholangitis [119].

**Fig. 2:**
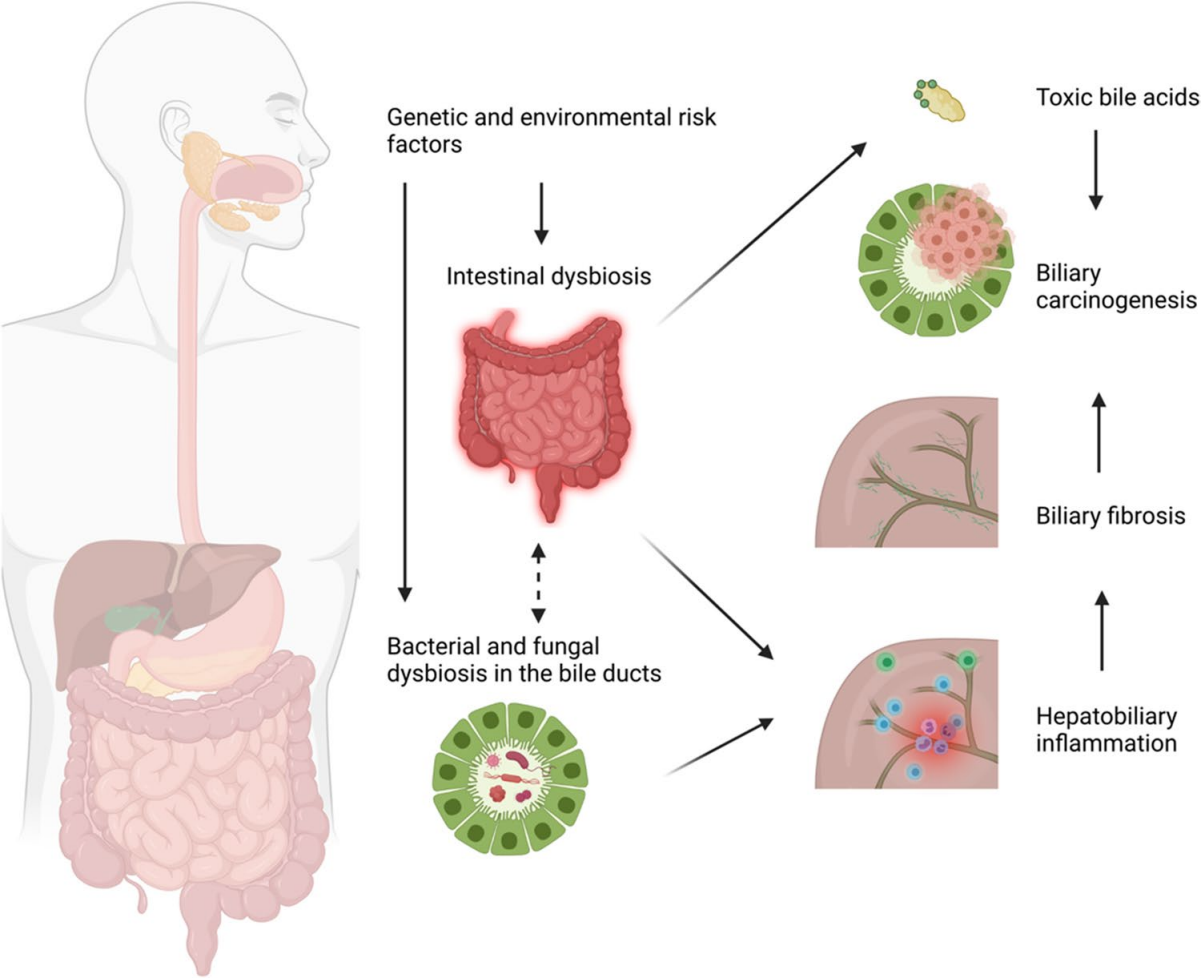
Hypothesized implication of biliary dysbiosis in primary sclerosing cholangitis. Environmental and genetic risk factors induce biliary dysbiosis directly or via intestinal dysbiosis. Bile duct dysbiosis leads to inflammation, breach of the biliary mucosal barrier, and potentially generating a toxic bile acid composition, which triggers bile duct fibrosis and cholangiocarcinogenesis

A fascinating new facet in PSC microbiota research is the role of the ductal bile microbiome (Table 4). Until recently, bile was considered a sterile fluid, but more recent reports show that a unique microbial ecosystem exists in subjects with and without hepatobiliary disorders [120]. Not only are cholestatic liver diseases associated with bile microbial alterations, but the bile microbiome also seems to vary by the etiology of cholestasis [121, 122]. A pilot study from Finland by Färkkilä and colleagues revealed minor to no microbiome alterations in PSC patients compared to healthy controls [123]. In contrast, a study conducted on a population from Northern Germany demonstrated an altered composition, reduced alpha diversity, and overrepresentation of certain pathobionts in the duodenal fluid of PSC patients compared to healthy subjects [124]. Dysbiosis was also observed in the upper alimentary tract and bile ducts, where the most significant differences were found compared to healthy controls. *Enterococcus faecalis*, a potentially pathogenic bacterium, showed the most robust increase in the ductal bile in PSC patients. The abundance of *Enterococcus* was strongly associated with a rise in the bile acid taurolithocholic acid, which is known to be pro-inflammatory and potentially carcinogenic (Fig. 2) [124]. A smaller report could reproduce some of the former study’s basic findings, such as altered bile microbiota composition and increased proportion of phylum Proteobacteria in PSC patients [121]. Although a fascinating subject, the study of ductal bile communities faces serious obstacles. Above all, bile sampling is an invasive procedure (usually requiring endoscopic retrograde cholangiopancreatography), limiting the availability in patients and even more in proper controls. On top of that, bile duct communities must be considered low-biomass microbiomes, which implies that their investigation is challenged by a high rate of false-positive signals resulting from contamination and sequencing-related biases and artifacts [125]. In defiance of these challenges, the engrossing hypothesis of bile duct pathology in PSC resulting from the breaking of tolerance to a dysbiotic biliary microbiome [4] makes it a subject worthy of further study.

**Table 4:** Overview of taxonomic alterations in case–control studies on the bile microbiota in PSC

Reference	Study population	Biological source	Sequencing technique	Enriched in PSC	Depleted in PSC
Tyc et al., 2020 [121]	5 PSC; 6 cholestatic controls without cholangitis*; 5 cholangitis patients*	Ductal bile	16S, V3–V4	Phylum: Proteobacteria	Phylum: Actinobacteria, Bacteroidetes, Firmicutes, and Fusobacteria
Liwinski et al., 2020 [124]	43 PSC, 22 cholestatic controls*	Ductal bile	16S, V1–V2	Phylum: Proteobacteria Genus level: *Enterococcus*, *Staphylococcus*, *Neisseria*, *Enhydrobacter*, *Prevotella*, *Lawsonella*, *Sphingomonas*, *Cutibacterium* Species level: *Enterococcus faecalis*, *Staphylococcus epidermidis*, *Streptococcus sanguinis*, *Enhydrobacter aerosaccus*, *Prevotella pallens*, *Veillonella dispar*	Genus level: *Collinsella*, *Clostridium* Species level: *Gemella sanguinis*, *Streprococcus gordonii*
Pereira et al., 2017 [123]	80 PSC (37 with early disease, 32 with advanced disease, and 11 with biliary dysplasia); 46 cholestatic controls*	Ductal bile	16S, V1–V3	Early-stage PSC vs controls: an unclassified *Clostridiales*, Otu00188, unclassified *Neisseriaceae* Otu00213) and one family, *Staphylococcaceae* Early stage PS.C vs advanced stage: genus *Streptococcus* and several *Streptococcus* OTUs	PSC with biliary dysplasia vs other: *Prevotella* OTU

*PSC*, primary sclerosing cholangitis.

To sum up, the stage of a cross-sectional description of intestinal taxonomic microbiome alterations in clinical PSC populations can be considered approximately completed. However, more extensive studies, ideally relying on shotgun sequencing and integrating multiple international cohorts, will reveal subtler changes. Now, prospective studies are warranted combining more functional information (through metagenomics, proteomics, metatranscriptomics, and metabolomics). The research of the bile microbiota in PSC is a promising though challenging area, and no knowledge is available on the mucosal microbiota of bile ducts. The study of viral communities in PSC will complete the picture of perturbations of the microecology in PSC.

## Evidence from animal models and potential biological mechanisms

As of the time, microbiota studies in autoimmune liver diseases have been mainly correlational. Even so, over recent years, researchers have yielded mechanistic support for a link between intestinal pathobionts and liver injury in autoimmune and cholestatic liver diseases. Together these studies support the concept of microbiome community alterations, bacterial translocation across the gut barrier, and amplified immune response in autoimmune liver diseases, though more nuanced pathophysiological aspects now come to light as well.

### Autoimmune hepatitis

In an elegant study, Manfredo Vieira et al. have demonstrated that translocation of the pathobiont *Enterococcus gallinarum* from the gut to the liver triggers autoimmune responses in humans and mice predisposed to autoimmunity. *E. gallinarum* DNA was detected in liver biopsy specimens from AIH patients. Coculturing *E. gallinarum* with human hepatocytes induced the autoantigens endogenous retrovirus glycoprotein 70 and β_2_-glycoprotein I, which are considered autoimmunity-promoting components [126]. Another recent study has shown that *Bifidobacterium animalis* ssp. lactis 420 (B420) alleviates S100-induced experimental autoimmune hepatitis (EAH) and modulates the gut microbiota configuration in mice. B420 decreased circulating endotoxin levels, suppressed the RIP3 signaling pathway in hepatic macrophages, and mitigated the proliferation of Th17 cells [127]. Zhang et al. reported that AIH patients feature increased gut permeability and RIP3 activation of hepatic macrophages. In mice, intestinal barrier dysfunction led to intestinal bacterial translocation, thus amplifying the hepatic RIP3-mediated innate immune response. Furthermore, GSK872 attenuated RIP3 activation and thus decreased the activation and accumulation of macrophages in the liver. Broad-spectrum antibiotic treatment resulted in reduced RIP3 activation and ameliorated liver injury [128]. Certain neurotransmitters have recently been proposed to regulate immune responses and play a critical role in autoimmunity. Xue et al. have shown that depletion of dopaminergic neurons promotes activation of hepatic iNKT cells and promotes concanavalin A (Con A)-induced liver injury (a mouse model of AIH) [129]. Ablation of the gut microbiota by an antibiotic mix reduced intestinal dopamine synthesis, and in turn, aggravated liver injury. The liver damage could be reversed by either restoring the gut microbiota or supplementation of a D1-like receptor agonist. These results point towards a regulatory axis encompassing the gut microbiome, nervous system, and immune system, which plays an essential role in autoinflammatory liver injury [129]. Moreover, dysfunctional bile acid synthesis and reduced farnesoid X receptor (FXR) activation were reported in AIH [130]. A recent study demonstrates that reduced commensal butyrate synthesis is implicated in hepatitis in FXR KO mice. Butyrate supplementation reversed dysregulated BA synthesis and mitigated liver inflammation in this mouse model [130].

### Primary biliary cholangitis

The E2 component of the mammalian pyruvate dehydrogenase complex (PDC-E2) displays a remarkable structural similarity with its bacterial counterpart. Therefore, it has been hypothesized that bacterially triggered breach of tolerance to PDC-E2 may be the first step in the pathogenesis of PBC [131]. Non-obese diabetic (NOD).B6 Idd10/Idd18 mice infected with *Novosphingobium aromaticivorans* or *E. coli* manifest liver lesions similar to the PBC’s pathology in humans [131, 132]. In mice, frequent injections with the bacterium *Streptococcus intermedius* resulted in PBC-like non-putrid cholangitis and an increase of circulating anti-gp210 (a crucial antibody in a subgroup of PBC patients); in the same mouse model, portal inflammation was induced in RAG2(− / −) mice by transferring splenic cells from *S. intermedius*–inoculated C57BL/6 wild-type mice [133].

Ma et al. reported that dnTGFβRII mice, a well-characterized murine model of PBC, show an altered composition of their intestinal microbiome, and administration of antibiotics mitigated hepatic T cell infiltration and bile duct lesions in these mice. Toll-like receptor 2 (TLR2)–deficient dnTGFβRII mice showed a more severe cholangitis activity correlated with disrupted epithelial barrier integrity [134].

Isaacs-Ten et al. have demonstrated that murine cholestasis alone does not cause liver injury in germ-free mice [135]. The authors have shown that endotoxin increases hepatocytes’ susceptibility to cell death following bile acid challenge. Macrophages promote intestinal leakiness and gut microbiome alterations during cholestasis by activating the inflammasome, leading to increased hepatic endotoxin exposure [135].

### Primary sclerosing cholangitis

Multidrug resistance 2 knockout (mdr2(− / −)) mice manifest a PSC-like liver disease phenotype. Interestingly, germ-free mdr2(− / −) mice display a more vigorous disease activity and cholangiocyte senescence, pointing towards a protective role of specific microbial consortia and their metabolites [136]. Tedesco et al. have demonstrated that livers from mdr2(− / −) mice harbor increased numbers of IL17A + γδTCR + cell populations, and that mdr2(− / −) mice display an increased intestinal abundance of *Lactobacillus* [137]. Furthermore, the authors found that the manifestation of hepatic inflammation and fibrosis in mdr2(− / −) mice was dependent on intrahepatic activation of γδ TCR + cells and expression of IL-17; these events were triggered by exposure to *Lactobacillus gasseri* [137]. In another study, mdr2(− / −) mice displayed an altered intestinal microbiota composition and NLRP3 inflammasome activation within the gut-liver axis. Intestinal dysbiosis in these mice correlated with intestinal barrier disruption and bacterial translocation, fueling the NLRP3-mediated immune response in the liver [138]. Thus, the microbiota seems to play a double-edged role in this mouse model featuring both protective and damaging effects.

Fuchs et al. have shown that the bile acid sequestrant colesevelam enhanced bile acid conversion by the microbiota towards secondary bile acids, thereby stimulating the secretion of GLP-1 from enteroendocrine L cells and attenuating liver and bile duct injury in mdr2(− / −) mice [139].

In a recent study incorporating human and murine specimens, Nakamoto et al. detected *Klebsiella pneumoniae* in fecal samples from patients with PSC. They demonstrated that certain *K. pneumoniae* strains disrupt the epithelial barrier resulting in translocation of *K. pneumoniae*, *Proteus mirabilis*, and *Enterococcus gallinarum*; the hepatic translocation of these pathobionts instigates bile duct damage and liver inflammation [140]. Furthermore, germ-free mice colonized with PSC patients’ fecal microbiota exhibited hepatic Th17 cell immune responses and an increased susceptibility to hepatobiliary injury, which could be mitigated by administering a RAR-related orphan receptor-γt (ROR-γt) inverse agonist [140].

To summarize, these studies support a causal relationship between gut microbes and autoimmune liver diseases and represent progress towards clinical application. However, it is imperative to recall that one cannot directly extrapolate the findings from these animal models to human patient populations, especially since no mouse model can fully recapitulate all essential features of any autoimmune liver disease [141]. Also, inbred mice do not reflect the genetic heterogeneity of humans. Moreover, experiments in wildling mice that harbor a microbiota more akin to humans on a variable genetic background are required to circumvent these shortcomings [142]. Furthermore, none of these studies thus far has drawn a complete and coherent causal trail between the microbiome and the clinical manifestations of autoimmune liver disease.

## Potential cues for microbiome-targeted therapies

**Fig. 3:**
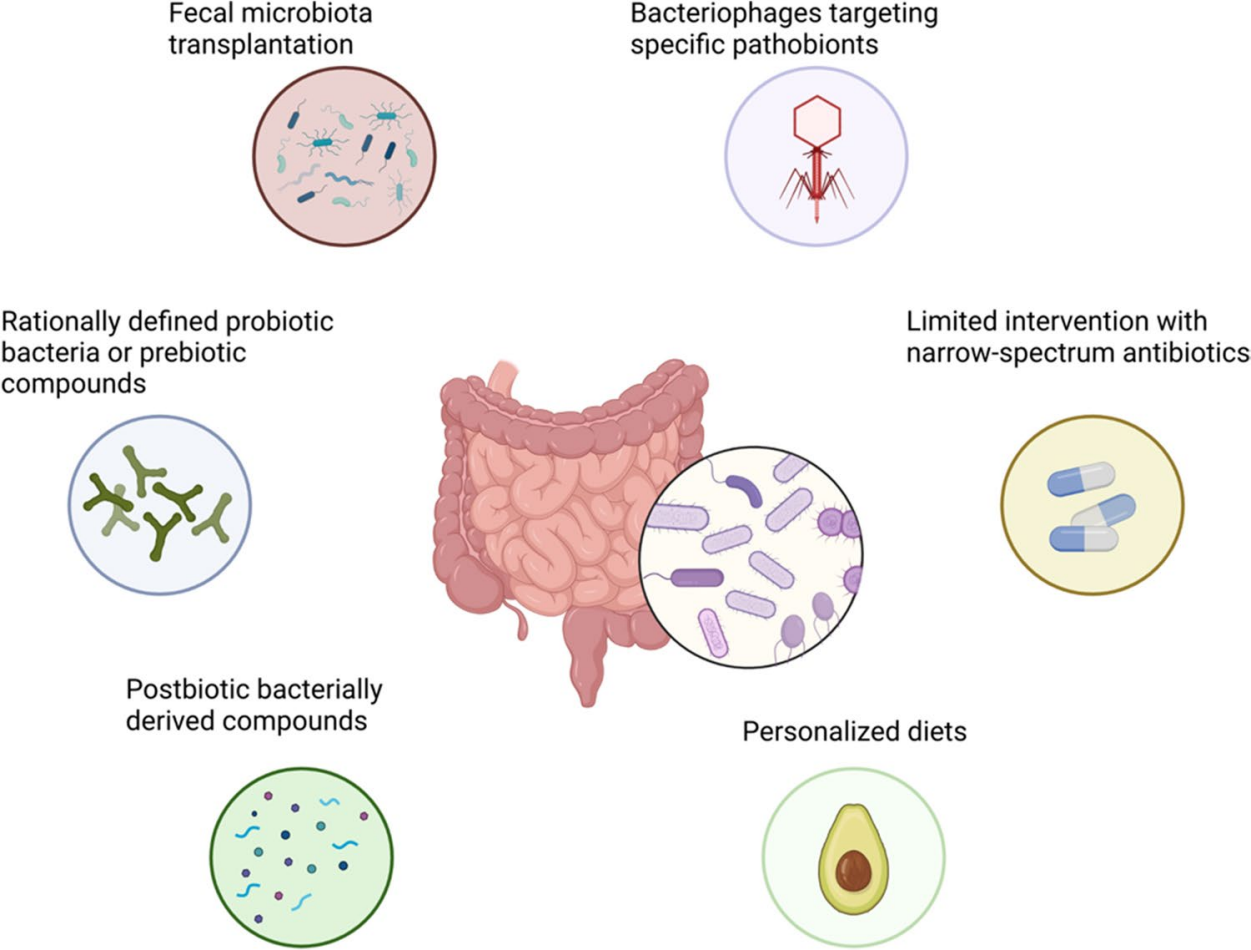
Microbiome-targeted therapeutic interventions

The swift advancement in microbiome research uncovered novel therapeutic avenues and reinvigorated “new old tools” targeting the microbiota for medical purposes (Fig. 3).

Interest in the therapeutic potential of fecal microbiota transplantation (FMT) has increased in recent years, testified by a sequence of randomized controlled trials (RCTs) in recurrent *Clostridioides difficile* infection (CDI) [143]. While a recent small trial indicated the potential benefit of FMT in PSC patients, large definitive RCTs are awaited [78]. It must be noted that the mechanism of FMT’s action is entirely unclear, and there are strong indicators that the engraftment of living microorganisms may not even be necessary [144]. Moreover, the potential risk of transferring uncharted harmful microorganisms raises safety issues [145]. Deciphering the mechanisms underlying FMT efficacy will yield more refined, more potent, and safer tools. Such a tool may be the engraftment of rationally defined bacterial strains. The idea of consuming live microorganisms to confer a health benefit is (so-called probiotics) is not new. This practice has been widespread for well over a hundred years. However, contradictory clinical evidence for their efficacy and lack of understanding of the mechanisms of action have hampered probiotics’ establishment in evidence-based medicine [146]. Nevertheless, the current next-generation sequencing era has provided researchers for the first time with the tools to thoroughly characterize the beneficial potential of microorganisms and harness their therapeutic potential. Such a suitable candidate bacterium could be the butyrate-producing *Faecalibacterium prausnitzii* [147].

An even more efficient strategy could be directly administering microbe-derived bioactive compounds conferring a health benefit (so-called postbiotics) [148]. Novel tools are emerging for parsing the microbiota metabolome and uncovering relevant host-microbiota metabolome interactions [149].

The counterpart approach to replenishing beneficial microbes or metabolites is the depletion of harmful microorganisms. A simple strategy to achieve this is represented by antibiotics. Indeed, as reviewed above, there is substantial evidence that antibiotics, particularly vancomycin, may positively affect PSC outcomes [69]. Considering the emergence of antibiotic resistance and the detrimental effects of antibiotic treatment on the gut microbiome with potentially unfavorable long-term consequences, the broad-spectrum activity of chemical antibiotics is the principal disadvantage preventing large-scale antibiotic treatment of patients with autoimmune liver diseases. A valuable alternative could be the use of small viruses, bacteriophages, which target bacteria. Phage therapy, long overshadowed by chemical antibiotics, garners renewed interest in modern medicine [150]. Phages usually target bacteria with species, or even strain specificity. New animal models, case studies, and recent clinical trials yield encouraging results [150]. However, mechanistic and pre-clinical insights on phage-pathogen interactions and host-microbiota-phage dynamics are required to lay the basis for RCTs in chronic inflammatory diseases.

The data provided by Manfredo Vieira et al. suggest a potential utility of vaccination against pathogenic species in autoimmune conditions [126].

Diet has a profound impact on the gut microbiome. Personalized diets are an exciting new tool to edit an individual’s gut microbiome, and clinical trials in the field of metabolic diseases are currently conducted [151]. It is tempting to speculate that such an approach could be effective in autoimmune diseases.

Further research is required to establish the utility of the therapeutic approaches proposed. Fundamental limitations to data published so far include their short time frame and PSC’s lack of suitable surrogate endpoints correlating with clinically meaningful outcomes. The latter restriction impedes interventional trials in PSC in general.

## Conclusions and future perspectives

We conclude that patients with AIH, PBC, and PSC have altered gut microbiome. This observation may also apply to other digestive tract segments and in PSC to ductal bile. These alterations seem to be disease-specific, although the evidence on this question is not conclusive yet. The limitations of the clinical microbiome data available include the cross-sectional designs and the biases associated with PCR-based microbiome profiling. In general, metagenomic sequencing is limited by its inability to account for the functional activity of the community. To accurately analyze health-related outcomes associated with microbial configuration, it is essential to divert the attention from the isolated study of different data types to an integrative approach incorporating multiple covariables from several data domains, such as metatranscriptomics, metaproteomics, and metabolomics, ideally in a prospective manner.

Moreover, in classical infectious diseases, phenotypes are often associated with only a subset of strains within microbial clades. Future studies should incorporate newer tools for strain-level analysis in metagenomic sequencing data [152]. Of note, the widespread practice of surveying patients’ microbiomes for microorganisms with “altered relative abundance” may not turn out conducive to deciphering the microbiota’s impact on pathogenesis as we have witnessed that an enteric bacterium such as *Enterococcus gallinarum* may translocate and cause pathology in predisposed individuals without being overrepresented in intestinal specimens [126, 140].

Gut microbiota dysbiosis or specific pathobionts play a causal role in several hepatic autoinflammation and autoimmune cholangitis animal models. However, the link between the compelling results from murine studies and human pathophysiology is unclear. This uncertainty arises from substantial heterogeneity between the animal models available and the human pathogenesis. Nevertheless, these studies provide a quintessential starting point and strong rationale for further research. A translational approach such as the ones given by Kanai and colleagues [140] and Manfredo Vieira et al. [126] are apt examples to follow for future studies.

Microbiome configuration and immune responses are highly variable among human individuals and often a higher proportion of variance is explained by inter-individual variation than by disease state. This inherent variability and complexity represent a formidable challenge and an ambit for artificial intelligence and machine learning to decode individualized microbiome-health reciprocation [153].
